# Short-Term Efficacy of a Multi-Modal Intervention Program to Improve Custom-Made Footwear Use in People at High Risk of Diabetes-Related Foot Ulceration

**DOI:** 10.3390/jcm14113635

**Published:** 2025-05-22

**Authors:** Jaap J. Van Netten, Lisa E. Vossen, Faye M. Driebergen, Danne Wolthuis, Maarten J. M. Merkx, Sicco A. Bus

**Affiliations:** 1 Department of Rehabilitation Medicine, Amsterdam UMC, University of Amsterdam, Meibergdreef 9, 1105 AZ Amsterdam, The Netherlands; l.e.vossen@amsterdamumc.nl (L.E.V.); s.a.bus@amsterdamumc.nl (S.A.B.); 2 Amsterdam Movement Sciences, Rehabilitation & Development, Amsterdam UMC, University of Amsterdam, 1105 AZ Amsterdam, The Netherlands; 3 Department of Clinical and Development Psychology, University of Amsterdam, 1001 NK Amsterdam, The Netherlands; f.m.driebergen@amsterdamumc.nl (F.M.D.); d.wolthuis@amsterdamumc.nl (D.W.); m.merkx@hsk.nl (M.J.M.M.); 4 Mental Care Group, 3447 GN Woerden, The Netherlands

**Keywords:** diabetes mellitus, foot ulcer, shoes, adherence, prevention, education, motivational interviewing, footwear

## Abstract

**Background**: Wearing custom-made footwear is key in diabetes-related foot ulcer prevention. However, adhering to wearing footwear is challenging, in particular at home. Evidence-based interventions with proven effect are needed, but scarce. We developed a multi-modal intervention to improve custom-made footwear use, and investigated short-term efficacy. **Methods**: We used a multidisciplinary multiphase approach to develop a three-modality intervention: structured education, motivational interviewing, and custom-made indoor footwear. To assess efficacy, we measured mean 2-week wearing time of custom-made footwear with a validated sensor, at baseline and after three months (primary outcome), and in the two weeks directly before and after each modality was administered (secondary outcomes). We assessed differences between timepoints using within-subjects paired *t*-tests. **Results**: 53 participants with high risk for ulceration were included: 30 with low [<8 h/day] baseline adherence), 17% females, mean age 66 (SD: 10) years, all with peripheral neuropathy and a recent foot ulcer (mean time since healing: 6 (SD: 9) months). Wearing time increased non-significantly from 4.0 (SD: 2.5) at baseline to 5.5 (SD: 4.3) after three months in the low adherence group (*p* = 0.068); this was 11.9 (SD: 2.3) to 12.0 (SD: 2.8) in the high adherence group (*p* = 0.898). Following provision of indoor footwear, wearing time increased significantly for low baseline adherence (∆2.7 h/day (95% CI: 1.0–4.4; *p* = 0.004) and high baseline adherence (∆2.0 h/day (95% CI: 0.5–3.4; *p* = 0.010). Following structured education, wearing time increased non-significantly in those with low baseline adherence (∆1.0 h/day (95% CI: −0.2–2.2; *p* = 0.098). Following motivational interviewing, wearing time remained similar in both groups. **Conclusions**: The multi-modal intervention program combining structured education, motivational interviewing and custom-made indoor footwear did not result in a statistically significant improvement in the wearing time of custom-made footwear after three months. However, significant improvements followed the provision of indoor footwear, and clinically relevant improvements followed structured education in people with low adherence, providing avenues for implementation and research.

## 1. Introduction

Diabetes-related foot ulcers have a significant negative impact on quality of life, patient mobility, and life expectancy [1]. The prevention of diabetes-related foot ulcers is therefore important. A key treatment strategy for prevention is the provision of custom-made footwear [2]. Custom-made footwear includes biomechanical design features, such as a rocker profile, a transmetatarsal bar, and local deepening or padding, that help reduce peak plantar pressures [3]. This footwear prevents the foot from excessive mechanical load during weight-bearing activities that cause the skin to break down, and protects a person at high risk from getting a foot ulcer while weight-bearing [2]. However, such footwear is only effective when it is worn. Steps taken barefoot, in socks only or in other types of footwear immediately increase the mechanical load on the foot, and thereby the risk of ulceration [2]. Guidelines therefore recommend that people at high risk of foot ulceration wear custom-made footwear at all times [2]. However, high levels of adherence are challenging to achieve. Studies generally show footwear adherence to be around 60–70%, exposing patients to high loads for the remaining time [4,5,6,7,8,9,10,11]. A key question from a clinical and scientific perspective is how to improve these adherence levels. For this, interventions to effectively improve footwear adherence are urgently needed [12].

Unfortunately, a recent systematic review of the literature showed interventions to improve adherence to be scarce [12]. Three interventions were found in this review: structured education, motivational interviewing (MI) and indoor footwear [12]. For education, it was found that this may improve foot-specific self-care; however, footwear adherence was not included in the self-care investigated in these studies [12]. MI is a person-centred communication technique designed to strengthen an individual’s motivation for change [13]. However, both a pilot randomised controlled trial (RCT) and a full RCT found little to no effect of one session of MI on footwear adherence [14,15]. For custom-made indoor footwear, a non-controlled pilot study found that the provision of such indoor footwear improved footwear adherence [8], but larger and controlled studies to confirm this finding have not yet been performed. This systematic review, international guidelines on foot ulcer prevention, and a research agenda on custom-made footwear have recommend that more research is needed on interventions to improve footwear adherence [2,12,16].

Improving adherence requires behaviour change. Behaviour is influenced by a wide range of personal, social, and environmental factors. To effectively elicit behaviour change, interventions should incorporate multiple components that target these diverse behavioural determinants [17]. In addition, such interventions should be personalised, evidence-based, and feasible to implement [12]. Based on these principles, we developed a multi-modal intervention to improve adherence to wearing custom-made footwear. This intervention is currently being tested for effectiveness in an RCT, as described in our protocol paper [18].

In addition to the long-term effectiveness investigated in the RCT, the short-term efficacy effects of the multi-modal intervention can provide insights into the direct effects of each of the modalities. Additionally, to allow replication and potential implementation of this multi-modal intervention, a detailed description of the intervention is provided [19,20]. For interventions aiming to change behaviour, such a description should also include assessments of the behavioural components included in the intervention [19,20]. In this manuscript, we describe the components of our footwear adherence intervention in sufficient detail to allow replication and implementation. Our aim was to investigate its short-term efficacy on custom-made footwear use.

## 2. Materials and Methods

### 2.1. Study Design

The data in this manuscript were obtained as part of the DIASSIST trial, an RCT on personalised prevention of foot ulcer recurrence [18]. The study was approved by the local ethics committee of Amsterdam UMC (reference number NL78943.018.21) and conducted in accordance with the Declaration of Helsinki. All participants gave informed consent before participating. Only participants in the enhanced therapy arm of this RCT were included in the current analyses. Therefore, the study design of this manuscript is considered an observational before–after cohort study design.

#### Development of the Intervention

This intervention is part of the personalised assistive device approach named DIASSIST. The DIASSIST approach, including the current intervention, was designed using a multiphase and multidisciplinary approach, described in detail in the full protocol [18]. In short, we: (i) assessed current evidence and (ii) (inter)national guidelines; (iii) designed the intervention; (iv) discussed this with 22 experts from different disciplines; (v) pilot-tested it with three patients; and (vi) finalised the intervention based on the lessons learned. The intervention consisted of modalities to improve footwear biomechanics, footwear adherence, and self-monitoring. In this manuscript, we focus on the footwear adherence-improving intervention only. This consisted of three modalities, the three interventions found in a systematic review and described in the introduction [12]: structured education, MI, and custom-made indoor footwear. These are described in detail in Section 2.3 (procedures).

### 2.2. Participants and Setting

Participants were diagnosed with diabetes mellitus type 1 or 2 and peripheral neuropathy, at least 18 years old and were all in possession of custom-made footwear (i.e., fully- or semi-custom-made footwear or off-the-shelf footwear with orthopaedic adjustments). All participants had healed from a plantar foot ulcer or (partial) foot amputation in the preceding four years, which characterized them as high risk for ulceration (International Working Group on the Diabetic Foot risk 3) [2].

Exclusion criteria were the presence of a foot ulcer, foot infection, active Charcot neuroarthropathy, bilateral amputation proximal to the metatarsal bones, or the inability to participate based on the treating physicians’ opinion. Written informed consent was obtained from all participants prior to inclusion.

Participants were recruited from the multidisciplinary foot clinics of five Dutch hospitals (one academic and four community-based hospitals). The treating rehabilitation physician or podiatrist identified and informed potential participants. When interested, the research team performed all subsequent communications before and during the study. Power calculations were conducted for the primary outcomes of the RCT, as described in the published protocol [18]; for the current efficacy study, no power calculations were performed.

### 2.3. Procedures

All participants in the DIASSIST RCT underwent a baseline screening as described in detail in our protocol [18]. The baseline screening consisted of obtaining demographic and clinical characteristics using assessments, measurements and questionnaires. Clinical assessment was performed by a trained researcher (LV). Measurements consisted of barefoot and in-shoe plantar pressure measurements using the valid EMED-X and Pedar-X systems (novel GmbH, Munich, Germany) [21,22], and physical activity measurements during seven consecutive days following the baseline visit using the valid MoveMonitor (McRoberts, The Hague, The Netherlands) [23]. Questionnaires consisted of the EuroQol 5D-5L (EQ5D5L [24]), the 36-item Short-Form survey (SF-36) [25] and two study-specific questions related to the feeling of a participant towards re-ulcerating and an assessment of their own risk of re-ulceration, both scored on a visual analogue scale ranging from 0 (not bad/no risk) to 100 (very/high risk).

To measure the primary outcome, wearing time of custom-made footwear, quantitatively and objectively, a validated wearing time sensor (Orthotimer, Balingen, Germany) was built in the insole of up to three pairs of participants’ regular custom-made footwear, and in the custom-made indoor footwear [26]. Wearing time data were obtained by reading out the sensor at the randomisation visit and at the study visit after three months, and by using the Groningen algorithm version 2 to determine daily wearing time [27].

Participants were randomised to either enhanced therapy or usual care at the randomisation visit, two weeks after baseline screening. Participants randomised to enhanced therapy were included in the analyses described in this manuscript and subsequently followed the intervention procedures for the three modalities.

#### 2.3.1. Modality 1: Structured and Personalised Education

**Figure 1:**
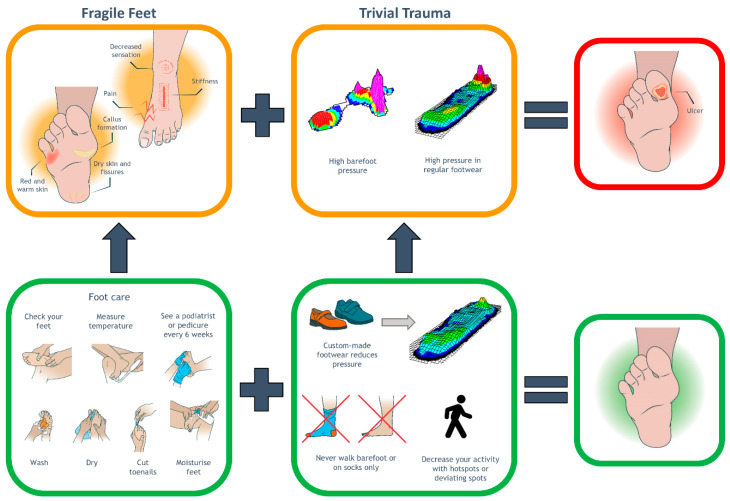
Example of the personalised Fragile Feet–Trivial Trauma educational leaflet (front page).

Various educational approaches exist to improve footwear adherence; however, evidence regarding the most effective approach is lacking. It is known that education in general is more effective when it is personalised, provided with a positive approach, in a first-person narrative, and when patients are asked to repeat it in their own words, i.e., the teach-back method [28,29,30]. Based on these principles, we designed a single structured and personalised education session. The content of this session was based on the Fragile Feet–Trivial Trauma (FF–TT) model for structured foot ulcer prevention education [31]. This is one of the few published educational models in foot ulcer prevention, and was chosen because it allows for adaptation to study-specific or local situations, and includes the potential for visual illustrations to explain multiple risk factors, as well as the interventions present to reduce these risks. Our adaptation of the model is shown in Figure 1, and an English version of the protocol (unilaterally translated by the authors) is provided in Supplementary Material S1. During this session, delivered at the randomisation visit at the start of the study, a trained researcher (LV) took a printed copy of the adapted FF–TT model and sat down with the participant. The researcher explained the content in the four boxes of the model, personalised to the participant’s situation and allowing the participant to ask questions at all times. After discussing all parts, with an emphasis on adherence to wearing custom-made footwear, the participant summarised this information in their own words. We did not measure the time, but the experience of the researchers indicated this took 5–10 minutes.

#### 2.3.2. Modality 2: Motivational Interviewing (MI)

MI is a communication method designed by Miller and Rolnick [13]. MI conversations follow a structured protocol with a defined aim: changing people’s behaviour by strengthening personal motivation and/or commitment to change. MI is a collaborative conversational style, aiming to elicit the client to verbalise their own arguments for changing their behaviour [13]. A basic premise of MI is the notion from Blaise Pascal that “people are usually more convinced by reasons they discovered themselves than by those found by others” [32]. There were two possible aims for the MI conversations in this study, depending on the baseline adherence of a participant. When baseline adherence was low (<80% of all steps prescribed footwear worn or mean wearing time < 8 h/day, cut-off criteria based on [5]), the aim of the MI session was to increase people’s adherence to wearing their custom-made footwear, i.e., setting a change goal. When baseline adherence was high (≥80% of all steps or ≥8 h/day), the aim was to maintain high adherence, i.e., setting a commitment goal.

The study-specific MI-protocol was designed by two researchers with >10 years of experience in footwear adherence studies (JvN and SB) and a clinical psychologist with >10 years of experience in MI (MM). The protocol followed the four stages of MI (Engage-Focus-Evoke-Plan). Feedback on footwear adherence was provided based on quantitative measurements of wearing time. All conversations were held in Dutch. An English version of the protocol (unilaterally translated by the authors) is provided in Supplementary Material S2.

All MI sessions were conducted by two researchers (JvN and SB). Both researchers received 10 h of training in MI before and an additional 8 h of follow-up training at quarterly intervals during the study by an experienced psychologist (MM). During the initial training, the principles of MI were taught and discussed in depth, and role play was used to practice. During the follow-up training, feedback was provided on recordings of MI sessions, additionally resulting in reflection on and enhancement of MI practice. The training and supervision content was in line with other studies for diabetes healthcare professionals, and the hours spent on training and supervision were higher than the mean in other studies [33].

Initially, the MI sessions were scheduled as home visits. However, after three such home visit MI sessions, it was decided that all subsequent MI sessions would be conducted by telephone, to reduce time required and to allow more effective future implementation. This decision was made together with the research team and external experts involved in the protocol development (see [18]), including patient representatives and an implementation expert, and justified by evidence from studies showing no difference between face-to-face or telephone-based MI [34,35].

A total of four telephone-based MI sessions were scheduled in the study [18]. The first MI session was held approximately two weeks after the randomisation visit. The MI sessions were recorded on a computer for training and analysis purposes. The recording was successfully made in 75% of the sessions (37 of 49), with 25% of sessions not recorded (recording program crash or technically unsuccessful recordings). The average duration of the recorded sessions was 20.3 (SD: 7.1) minutes. Additional MI sessions were scheduled after 3, 6 and 9 months in the study [18]; however, these are outside the scope of the current manuscript.

To assess treatment fidelity and quality of the MI in line with international recommendations [36], at least 20% of all of the MI sessions (including those at 3, 6 and 9 months) were randomly selected and assessed by a trained assessor (FD or DW). Recordings with a minimum duration of 18 min were scored using the Dutch version of the Motivational Interviewing Treatment Integrity (MITI 4) code [37]. Eight randomly selected recordings (including two of the first MI session) were scored by both assessors and showed high agreement (intraclass correlation [two-way mixed effect models for absolute agreement of average measures] > 0.93 for all 16 items that were assessed).

#### 2.3.3. Modality 3: Custom-Made Indoor Footwear

After the structured education at the randomization visit, participants were provided with the choice of receiving custom-made indoor footwear. The indoor footwear was an addition to their current regular custom-made footwear, designed to be worn indoors and aiming to improve footwear adherence indoors by addressing patient-reported reasons for not wearing the regular custom-made footwear indoors, such as heavy weight, difficulty to don and doff, warm feet, dirty from outdoor use [38]. This footwear was provided free of charge, with the average production costs of EUR 385 covered by the study funding. Participants could accept outright, reject outright (e.g., because they were already in possession of indoor footwear, or because they did not see a need), or delay the decision. When delayed, the choice would be given again at the study visit after three months. If rejected, the choice would not be offered again. When the participant accepted, production of the indoor footwear was immediately started.

The custom-made indoor footwear was provided according to the protocol described in detail in our study regarding its design and development [38]. In short, the shoe-last of the existing custom-made footwear was used, the midsole and outsole design elements were similar to those of the regular custom-made footwear, while the upper consisted of soft materials (felt or microfiber—participant’s choice) and a Velcro fastener and zipper were used for shoe closure. It was aimed to deliver the indoor footwear within 6 weeks, but the actual delivery time was a mean 14 (SD: 6) weeks. Upon delivery, in-shoe plantar pressure while walking was checked and improvements made when needed, according to our protocol [18]. When satisfactory, participants were provided with the indoor footwear and allowed to wear it immediately inside their house, with no further instructions or restrictions.

### 2.4. Outcomes

The primary outcome was the difference in mean daily wearing time between the two weeks before the 3-month study visit and the two weeks before randomization. This was defined as the hours per day during which any pair of custom-made footwear was worn. A minimum of five days of valid wearing time measurements were required for a period to be considered a valid outcome measurement [23].

Secondary outcomes were: (i) a per-protocol analysis of the primary outcome, in participants who successfully followed all three intervention modalities; (ii) the difference in wearing time between the two weeks preceding and following the structured education session; (iii) the difference in wearing time between the two weeks preceding and following the MI session; (iv) the difference in wearing time between the two weeks preceding and following provision of the custom-made indoor footwear.

The following scores were obtained for MI-fidelity and interpreted using the MITI 4 guidelines [37]: (a) the relational global score, composed of scores for partnership and empathy; (b) the technical global score composed of scores of evoking change talk and softening sustain talk; (c) the mean percentage of complex reflection; (d) the mean reflection-to-question ratio; (e) the mean total MI-consistent behaviours.

The demographic and clinical characteristics as obtained during the baseline visit were investigated as outcome measures to investigate potential explanatory variables associated with the primary outcome (wearing time difference) and with all three secondary outcomes. Additionally, MI quality (based on MITI-scores) was associated with the secondary outcome “iii”.

Finally, we assessed the behavioural components included in the three modalities. This assessment was based on the COM-B model (Capability, Opportunities, Motivation-Behaviour) [17]. We evaluated the presence of the six behaviour sources and nine intervention functions in this model [17]. We then used the theories and techniques taxonomy tool to identify the behaviour change techniques applied (from a list of 76), and the modes of action applied (from a list of 26) [39]. This qualitative assessment was performed by one author (JvN), checked by two authors (FD and DW), and discussed until consensus was reached among all three authors.

### 2.5. Statistical Analyses

Only available data were used in statistical analyses; missing data were not imputed. Intention-to-treat was used for the primary outcome, and statistically tested using a within-subjects paired *t*-test, comparing mean daily wearing time at three months versus at baseline, with alpha = 0.05. Secondary outcomes were tested using a within-subjects paired *t*-test with alpha = 0.05 and Bonferroni–Holmes correction for multiple testing.

To investigate the association between participant characteristics and change in wearing time, we created a dichotomous variable (wearing decreased/increased), based on the change in wearing time between baseline and three months. We univariately tested the association of all participant characteristics with these variables using Pearson’s correlation or Student’s *t*-tests. All characteristics with a *p*-value < 0.15 were included in multivariate analyses. For multivariate analyses, we performed a logistic regression, using forward likelihood ratio as the entering method for all variables that were identified in univariate analyses. The variables included in the final regression model, their odds ratio (95% CI) and *p*-value, and the model’s Nagelkerke R^2^ were reported.

## 3. Results

### 3.1. Baseline Characteristics

**Figure 2:**
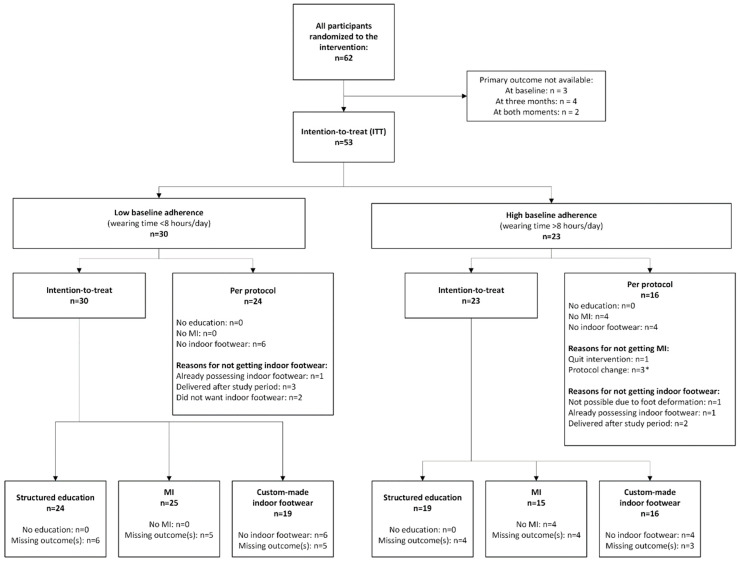
Flowchart of study participants. *: protocol change (from face-to-face to telephone conversations) as described in the methods.

Of the 126 participants in the DIASSIST RCT, 62 were randomized to the enhanced therapy group, and complete wearing time (primary outcome) data were available of 53 (Figure 2). Their characteristics, similar to other studies in this population [12], are described in Table 1. Of the 53 included participants, 30 (57%) had low baseline adherence (Figure 2). A total 40 participants (75%) completed all three intervention modalities according to protocol, 24 with low baseline adherence and 16 with high baseline adherence (Figure 2).

### 3.2. Primary and Secondary Outcomes

**Figure 3:**
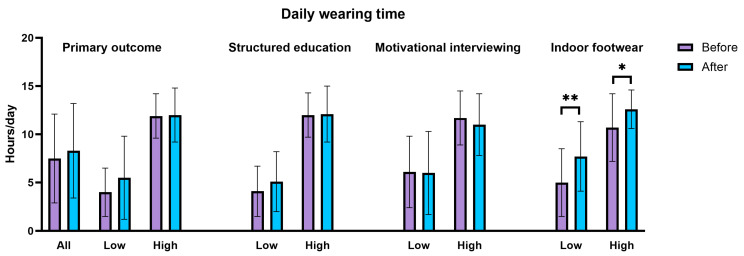
Daily wearing time of custom-made footwear before and after the intervention (intention-to-treat) and before and after each modality. Note: low = low baseline adherence; high = high baseline adherence; *: *p* = 0.010; **: *p* = 0.004.

Mean daily wearing time improved non-significantly by 0.9 h (*p* = 0.095; 95% CI: −0.2–1.9) (Table 2 and Figure 3). Subgroup analyses separated for baseline adherence levels showed an increase from 4.0 to 5.5 h/day in those with low baseline adherence (*p* = 0.068), and a sustained wearing time (11.9 to 12.0 h/day) in those with high baseline adherence (*p* = 0.898).

Secondary outcomes showed statistically significant increases in wearing time following provision of the indoor footwear in both those with low and high adherence levels at baseline, while structured education and the first MI session did not result in significant increases in wearing time (Table 2).

**Figure 4:**
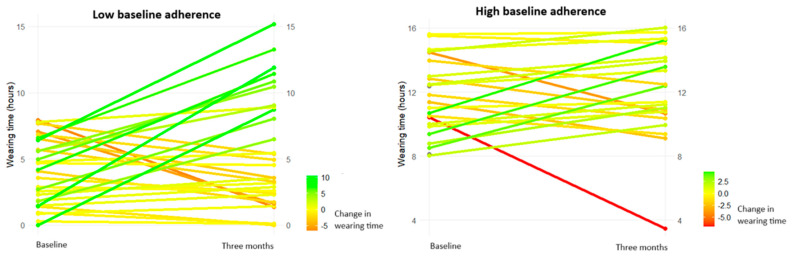
Changes in daily wearing time of custom-made footwear.

Visual representations of the changes in wearing time are shown in Figure 4. Of those with low baseline adherence, 57% (n = 17) increased their wearing time, with 28% (n = 9) showing an increase of at least 4 h. In those with high baseline adherence, 56% (n = 14) sustained or increased their wearing time, while one participant dropped their wearing time below 8 h/day (Figure 3).

### 3.3. Assessment of MI-Fidelity

Of the 49 MI sessions, 20 (41%) were > 18 min, and were assessed for fidelity (Table 3). Of these, twelve sessions were with people with low baseline adherence and aimed to change behaviour, and eight sessions with people with high baseline adherence and aimed at sustaining healthy behaviour. In sessions aimed at behaviour change, MI-consistent behaviour was lower, as a result of lower scores for “softening sustain talk” and for “providing affirmation”, and higher scores for “giving of information”. No associations were found with any of the scores and the change in wearing time in the two-week period following the MI. For comparison, MITI-scores from podiatrists trained in two other studies where MI was applied in diabetes-related foot disease are added (to be discussed in Section 4).

### 3.4. Factors Associated with Increased Wearing Time

For the group with low baseline adherence, we found that never smoking, a higher assessment of their own risk of re-ulcerating and a lower quality of life (general health) were associated with an increase in wearing time (*p* < 0.15) in univariate analyses. In multivariate analyses, personal risk assessment (ranging from 0 to 100) remained, with an odds ratio of 1.03 (95% CI: 0.999–1.063; *p* = 0.059), and R^2^ of the model 0.209.

For the group with high baseline adherence, we found that a higher HbA1c, lower quality of life (emotional well-being) and lower baseline adherence outdoors were associated with an increase in wearing time (*p* < 0.15) in univariate analyses. In multivariate analysis, lower quality of life (emotional well-being) remained, with an odds ratio of 1.19 (95% CI: 0.994–1.411; *p* = 0.058), and R^2^ of the model 0.475.

### 3.5. Assessment of the Behavioural Components of the Intervention

The behaviour sources, intervention functions, behaviour change techniques and modes of action differed between the three modalities (Supplementary Material S3). Structured education had the fewest in each category, while MI had the most. MI potentially includes up to eighteen different change techniques, but their application was not included in all MI sessions (see also Table 3). Custom-made indoor footwear applied different behavioural components compared to structured education and MI, with the latter two targeting psychological capabilities and reflective motivation as behaviour sources, while indoor footwear targets physical capabilities and physical opportunities. For a detailed assessment of all components, see Supplementary Material S3.

## 4. Discussion

The footwear-adherence-improving intervention investigated here is the most extensive of its kind to date. It was a multi-modal intervention, combining three different modalities that could potentially improve footwear adherence, with multiple behavioural strategies incorporated. To our surprise, this intervention did not significantly improve footwear adherence, measured as mean daily wearing time, between baseline and three-month follow-up. However, we did find that the majority of participants increased their adherence, or sustained their high adherence, and that wearing time increased significantly immediately after the provision of custom-made indoor footwear. These results provide avenues for further research and for implementation in clinical practice.

### 4.1. Modality 1: Structured and Personalised Education

The structured education provided in this study was short, easy to deliver, and will be cheap to implement. It only requires adaptation of the FF–TT model to a local situation, a printed sheet, and 5–10 minutes of an educator’s time. While statistically non-significant, the immediate change in wearing time from four to five hours/day in the group with low baseline adherence following this education was positive. Any improvement in footwear adherence can be clinically meaningful, and with education so cheap and easy to perform, we suggest implementing this in daily clinical practice. We did not assess the effects of repeating this structured education in this study, which could possibly further increase the wearing time.

### 4.2. Modality 2: Motivational Interviewing (MI)

MI did not result in changes in footwear adherence. This is similar to the only RCT on this intervention [15], but differs from the pilot RCT, where short-term changes were shown [14]. We suggest that this latter difference is explained by the already achieved effect of improving footwear adherence following the structured education in our study, as the educational aspect of MI may have driven the short-term improvements in that pilot RCT. Furthermore, the wearing time before the MI session was already higher than after the structured education session. Since both modalities include educational and knowledge components, the first potential gains using these components had already been achieved after the structured education was provided.

A second explanation for a lack of behaviour change following MI is related to the multi-modality of the intervention. Footwear adherence was especially low inside the house. However, when discussing this according to the MI-protocol, many participants indicated that they would change this as soon as their indoor footwear was provided, which was scheduled a few weeks after the MI session. That made it harder to motivate participants to change their behaviour immediately following the MI session, as they perceived less necessity to change behaviour for only a few weeks. However, this could have led to multiple weeks of lower adherence, since the delivery of the indoor footwear was delayed in some participants. New strategies to generate short-term behaviour change during MI sessions are therefore important to cover this ‘waiting period’.

A third explanation is that MI may not be effective in improving footwear adherence in this population [15]. Contrary to other behaviours where MI is used (e.g., substance use), the negative effects of footwear non-adherence are only experienced on the longer-term (i.e., foot ulceration, hospitalisation and amputation), while the negative effects of adherence are felt in the short-term (i.e., having to wear custom-made footwear at all times, even when this is considered unpleasant). This is also known as the prevention paradox [42], which defines the difficulty of doing something to prevent an unwanted result, such as a foot ulcer. The low perceived risk of ulceration (average 37 on a scale of 0–100) and the association of a higher perceived risk with a higher likelihood of increasing wearing time suggest that participants may indeed not experience the long-term risk. Personal health beliefs and risk assessments may play a role in the potential efficacy of MI. Future research should investigate this, and MI protocols specifically targeting these psychological traits may need to be developed.

A fourth explanation concerns the efficacy of MI-provision by the two researchers. Both received adequate training in line with other studies [33], and their MI-fidelity was higher or similar compared to other studies, as shown in Table 3 [40,41,43]. However, the global scores suggest an average level, but not a good level. If MI had been applied by a person highly skilled in MI (e.g., an experienced psychologist), MI-fidelity might have been higher. While higher MI-fidelity does not automatically increase behavioural outcomes, it does increase the likelihood for the MI causal pathways to be effective and, in turn, facilitate behaviour change [44,45]. Furthermore, the researchers did not have a therapeutic alliance with the participants. A treating physician or podiatrist with a longstanding therapeutic alliance might achieve different effects, especially when that person is also highly skilled in MI.

Finally, we only included the short-term results in this manuscript, which entailed one MI session. MI may lead to more results in the longer term and when repeated. The potential benefits of repeated MI sessions have been suggested by others [14,15,19]. The DIASSIST trial, which includes four MI sessions for each participant, is still ongoing. Once completed, the results of multiple MI sessions will be analysed and reported.

For clinical practice, there seems to be no case to implement a single MI session to improve footwear adherence at the moment. However, the spirit of MI and the communication techniques incorporated in this technique are valuable methods for practitioners working with people with diabetes-related foot disease, as person-centred and positive communication between healthcare professionals and patients is associated with higher adherence to footwear [46] and other therapies [47,48]. For this, MI can still be an effective method. Additionally, multiple MI sessions or repeated use of MI in a consultation could still be effective, and remain to be investigated.

### 4.3. Modality 3: Custom-Made Indoor Footwear

The biggest change in footwear adherence was achieved with the provision of custom-made indoor footwear. In both people with low and high baseline adherence, a statistically significant 2 h increase was found after the provision of this indoor footwear compared with the preceding two weeks. This confirms results from an earlier non-controlled study, where a significant increase in adherence after providing custom-made indoor footwear was also found [8]. Indoor footwear is a different strategy than structured education or MI, as seen in the different behaviour sources and intervention functions it targets. Indoor footwear changes physical capabilities and opportunities, as well as the environmental context, and both appear to be key avenues to improve footwear adherence in this population. Participants also reported that having such indoor footwear made it much easier to break their habit of not wearing shoes inside the house. The indoor footwear was kept next to their bed (at night) or next to their door (when leaving the house), making it accessible. This aligns with findings from others that the storage location or accessibility of (custom-made) footwear is an important variable explaining footwear adherence [49].

The positive findings related to indoor footwear provision cannot be seen or analysed separately from the other modalities. As discussed above, both the structured education and the MI session prepared participants to start changing their behaviour and wear the indoor footwear once provided. Additionally, the longer-term wearing time outcomes are needed to completely interpret changes in footwear adherence. Wearing time immediately following indoor footwear provision was higher than at the three-month study visit (see Table 2), indicating that people might fall back into old habits with only short-term interventions. The ongoing DIASSIST trial will answer this question.

For clinical practice, we advocate the implementation of indoor footwear as an addition to regular footwear. The additional costs are, in our opinion, justified in relation to the 2 h increase found in footwear adherence and the positive patient experiences. We hope that the findings from this study will stimulate healthcare systems to start to reimburse indoor footwear, while footwear companies simultaneously make efforts to develop affordable, biomechanically safe indoor footwear.

### 4.4. Strengths and Limitations

A strength of this study was the multi-stakeholder approach to the design of this multi-modal intervention, with input from patients, healthcare professionals and researchers. Furthermore, analysing people with low baseline adherence separate from those with high baseline adherence was a strength, as they require a different educational approach and have different potential for improvement. Another strength was using objective instruments to quantitatively measure footwear wearing time [5]. However, this was also a limitation, as it resulted in missing values. When participants forgot to bring all their footwear to study visits or when study visits were delayed for medical or participant preference reasons, data were missing that could not be retrieved. We decided not to impute missing data, because wearing times were very time-specific, with the interventions provided at various moments during the three months of the study. Additionally, we chose to analyse the two weeks before and after the specific study visits to specifically capture the short-term effects. Continued analysis of the wearing time over the complete three months, and over the complete 12 months of the DIASSIST trial, will provide more insights into behaviour changes. Improvements in sensor batteries, in sensor storage capacity, in techniques to allow participants to read out sensors themselves, or automated synchronisation of sensor data to the cloud are needed to overcome the practical limitations in future research. Until then, researchers should be aware of the challenges related to longer-term continuous measurements of wearing time in footwear.

A combined strength and limitation of the study was the multi-modality of the intervention. It was a strength, as it includes different strategies, thereby allowing personalisation. At the same time, it was a limitation, as it is not possible to disentangle the effects of the different modalities. We have discussed some potential interpretations above, but these should be considered in light of this shortcoming.

A limitation in the application of MI was the use of two researchers. They were specifically trained and dedicated to these MI sessions, but that limits generalizability to daily clinical practice. However, training showed similar MI-fidelity scores compared to studies where podiatrists were trained [40,41,43], suggesting that the findings are generalisable to that clinical profession. As neither the researchers nor podiatrists were highly skilled in MI, including a psychologist in the multidisciplinary team for foot ulcer treatment and prevention could provide avenues for better implementation of MI, as also suggested by others [50,51].

A limitation in the provision of indoor footwear was the delays in its provision. Despite having dedicated footwear companies and using the existing regular custom-made footwear as the basis for the indoor footwear, the scheduled six-week timeframe for their production and delivery was frequently not met. The reasons were primarily logistical, showing the complexity of manufacturing custom-made footwear. With five participants (10%) not receiving their indoor footwear in the study period, and with indoor footwear the biggest driver of increased wearing time, the primary outcome might have shown a statistically significant increase in wearing time if they had received their indoor footwear in a timely manner.

Another limitation in the study was not formally assessing cognitive impairments of participants. However, in the structured education and in the MI session, participants were asked to explain their situation in their own words. These answers were not formally assessed, but we experienced that all participants were able to explain their situation at a basic (or higher) level of understanding.

Other confounding factors that may have influenced the results were investigated in multivariate analyses, with most demographic characteristics showing no association with change in wearing time. However, the numbers were too small to also investigate the association of these factors with, for example, MI-fidelity. We could therefore not assess if there was an association between patient characteristics and likelihood of successful MI. Larger studies are needed to identify potential intricacies and associations between the successful application of the interventions and patient characteristics.

Finally, the current manuscript is limited to a before–after observational design, and did not include a control group. As described above, the ongoing DIASSIST trial will publish the control group findings once available. However, as a detailed description of an intervention is crucial for both assessment and implementation, and often too long to be incorporated in an RCT paper, we decided to describe those in this manuscript. We hope that this will stimulate uptake and implementation.

### 4.5. Future Research

Immediate future research concerns the longer-term findings to be obtained in the ongoing DIASSIST trial, with footwear adherence outcomes measured up to 12 months after study inclusion. The RCT will also report clinical findings, including foot health status (e.g., foot ulcer recurrence). In addition, we suggest that indoor footwear should be separately investigated in an RCT, and that such an RCT should then also incorporate other populations (e.g., people with diabetes at moderate risk of ulceration, or people without diabetes at high risk of ulceration). Furthermore, other avenues to improve footwear adherence should still be pursued. More different types of footwear for other contexts can be considered, such as for summer, for swimming or for religious buildings [16]. As a first step, better predictors for footwear adherence should be found, as current studies only explain up to 20% of the behaviour [4,6,7,49,52]. As others have suggested, psychological, environmental and social factors should be included in such studies [6]. The finding in this study that personal risk assessment was associated with increased wearing time in people with low baseline adherence does suggest that illness perception could be a relevant psychological variable to investigate in more detail in such future studies. When better predictors are found, interventions specifically targeting the change in these predictors may be developed.

## 5. Conclusions

A multi-modal footwear adherence improving intervention combining structured education, motivational interviewing and custom-made indoor footwear did not result in statistically significant improvement of wearing time of custom-made footwear after three months. However, immediate significant improvements were seen following the provision of indoor footwear, and clinically relevant improvements following structured education in people with low adherence. We therefore suggest that structured and personalised education and custom-made indoor footwear are implemented in daily practice, while future research investigates longer-term outcomes and differences with a control population.

## Figures and Tables

**Table 1:** Baseline characteristics of the included participants.

Variable	N = 53
**General characteristics**	
Sex (female/male)	17% (9)/83% (44)
Age (years)	65.7 ± 10
BMI (kg/m^2^)	29.6 ± 5.7
Ethnicity (Caucasian)	94% (58)
Level of education	
Low	34% (18)
Medium	40% (21)
High	26% (14)
Living situation (alone/together)	28% (15)/72% (38)
Work situation (employed/unemployed)	30% (16)/70% (37)
Smoking	
Yes	9% (5)
No	32% (17)
History of smoking	59% (31)
Alcohol	
Yes	53% (28)
Special occasions	11% (6)
No	36% (19)
Use of a walking aid	32% (17)
Quality of life (EQ5D) ^a^	4.1 ± 0.6
Quality of life—physical functioning ^b^	53 ± 24
Quality of life—physical limitations ^b^	45 ± 42
Quality of life—emotional limitations ^b^	68 ± 39
Quality of life—energy and fatigue ^b^	57 ± 21
Quality of life—emotional wellbeing ^b^	77 ± 18
Quality of life—social functioning ^b^	72 ± 25
Quality of life—pain ^b^	68 ± 22
Quality of life—general health ^b^	46 ± 18
Daily steps	5246 ± 3538
Footwear adherence	64% ± 24%
Footwear adherence indoor	57% ± 29%
Footwear adherence outdoor	77% ± 27%
**Diabetes-related characteristics**	
Type of diabetes (1/2)	17% (9)/83% (44)
Duration of diabetes (years)	19.1 ± 11
HbA1c (mmol/mol)	60.5 ± 18
Neuropathy	100% (53)
Inability to feel 10 g monofilament	96% (51)
Inability to feel tuning fork	94% (50)
**Comorbidities**	
Intermittent claudication	28% (15)
Retinopathy	77% (41)
Laser therapy	24% (10)
Nephropathy	38% (20)
Dialysis	4% (2)
**Foot-related characteristics**	
Time since healing last ulcer (months)	6.1 ± 9.3
History of Charcot	15% (8)
Amputations	
Absent	60% (32)
Digiti 2–5	11% (6)
Hallux	6% (3)
Metatarsal region	19 (10)
Forefoot or higher	4% (2)
Foot deformities ^c^	
Absent	0% (0)
Mild	6% (3)
Moderate	76% (40)
Severe	18% (10)
**Personal risk assessments**	
How bad would you feel if you get a new ulcer? ^d^	72 ± 27
How do you assess your risk of getting a new ulcer? ^d^	37 ± 28

Note: values are mean ± SD or % (n). ^a^: range from 1 (lowest) to 5 (highest). ^b^: range from 0 (lowest) to 100 (highest). ^c^: Foot deformities scored as described in the detail in the published protocol [18]. ^d^: Both behavioural questions were scored on a visual analogue scale, ranging from 0 (not bad/no risk) to 100 (very bad/high risk).

**Table 2:** Primary and secondary outcomes of daily wearing time of custom-made footwear.

	n	Before (Hours/Day)	After (Hours/Day)	Difference (95% CI)	*p*-Value
**Primary outcome (ITT)**					
All	53	7.5 (4.6)	8.3 (4.9)	0.9 (−0.2–1.9)	0.095
Low baseline adherence	30	4.0 (2.5)	5.5 (4.3)	1.5 (−0.1–3.1)	0.068
High baseline adherence	23	11.9 (2.3)	12.0 (2.8)	0.1 (−1.1–1.3)	0.898
**Primary outcome (PP)**					
Low baseline adherence	24	3.7 (2.4)	5.1 (3.9)	1.4 (−0.5–3.2)	0.136
High baseline adherence	16	11.9 (2.6)	12.5 (2.3)	0.6 (−0.8–1.9)	0.405
**Modality analyses**					
**Low baseline adherence**					
Structured education	24	4.1 (2.6)	5.1 (3.1)	1.0 (−0.2–2.2)	0.098
Motivational interviewing	25	6.1 (3.7)	6.0 (4.3)	−0.2 (−1.2–0.9)	0.763
Indoor footwear	19	5.0 (3.5)	7.7 (3.6)	2.7 (1.0–4.4)	0.004
**High baseline adherence**					
Structured education	19	12.0 (2.3)	12.1 (2.9)	0.0 (−0.9–0.9)	0.967
Motivational interviewing	15	11.7 (2.8)	11.0 (3.2)	−0.7 (−2.4–1.0)	0.383
Indoor footwear	16	10.7 (3.5)	12.6 (2.0)	2.0 (0.5–3.4)	0.010

Note: Values are mean (SD) daily wearing time of custom-made footwear in the periods before and after the intervention as defined in the methods, or mean (95% confidence interval). ITT = intention-to-treat; PP = per-protocol.

**Table 3:** Assessment of MI-fidelity and comparison with similar studies.

MITI Variable	DIASSIST			Comparison	
	All (n = 20)	Change (n = 12)	Sustain (n = 8)	AU (n = 11)	NL (n = 14)
**Global scores—technical** ^a^					
Change talk	3.9 (0.8)	3.8 (0.8)	3.9 (0.8)	3.1 (1.3)	3.2 (1.0)
Soften sustain	3.3 (0.6)	3.0 (0.6)	3.6 (0.5) *	1.4 (1.6)	3.0 (0.6)
**Global scores—relational** ^a^					
Partnership	3.0 (0.9)	2.8 (0.6)	3.3 (1.3)	3.1 (1.0)	2.7 (0.7)
Empathy	4.0 (0.7)	4.0 (0.5)	4.0 (0.9)	3.1 (1.0)	3.6 (0.8)
**Behaviour counts** ^b^					
Giving information	5.4 (3.1)	4.1 (2.0)	7.3 (3.5) *	13.4 (5.1)	2.9 (2.1)
Questions	16.7 (9.1)	18.0 (9.8)	14.8 (8.2)	18.9 (5.3)	13.8 (7.7)
Simple reflection	10.9 (4.6)	10.0 (4.3)	12.3 (4.9)	2.4 (1.7)	9.1 (5.6)
Complex reflection	7.8 (4.8)	8.1 (5.1)	7.2 (4.7)	1.1 (1.2)	3.0 (2.7)
Persuade with permission	0.8 (1.2)	0.8 (0.9)	0.9 (1.7)	0.1 (0.3)	0.6 (0.9)
**MI-adherent behaviour** ^b,c^					
Affirm	4.0 (1.9)	3.2 (1.6)	5.1 (1.9) *	0.6 (0.8)	3.8 (3.3)
Seeking collaboration	2.7 (2.2)	2.2 (1.8)	3.4 (2.7)	2.9 (2.3)	0.6 (0.9)
Emphasising autonomy	0.1 (0.4)	0 (0)	0.3 (0.7)	0.4 (0.6)	0.1 (0.3)
**MI non-adherent behaviour** ^b,d^					
Persuade	2.0 (1.6)	2.2 (1.9)	1.6 (1.2)	0.4 (0.7)	3.4 (2.7)
Confront	0.3 (0.7)	0.3 (0.5)	0.4 (1.0)	-	0.5 (1.0)
**MITI summary scores**					
Relational score ^a^	3.5 (0.7)	3.4 (0.5)	3.6 (1.0)	-	3.1 (0.7)
Technical score ^a^	3.6 (0.6)	3.4 (0.6)	3.8 (0.6)	-	3.1 (0.7)
Reflection:question ratio ^e^	1.5 (1.1)	1.2 (0.6)	2.0 (1.6)	-	1.0 (0.6)
% complex reflections ^f^	40 (17)	43 (20)	36 (13)	-	23 (15)
MI consistent ^b^	6.8 (2.9)	5.4 (1.8)	8.8 (3.1) **	-	4.4 (3.9)
MI inconsistent ^b^	2.3 (1.7)	2.4 (1.8)	2.0 (1.7)	-	3.9 (3.5)

Note: MITI = Motivational Interviewing Treatment Integrity (MITI 4). AU = Australian study on MI, reference: [40]; NL = study from the Netherlands on MI, reference: [41]. ^a^: Scores ranging from 1 (lowest) to 5 (highest). ^b^: Scores are the number of times the behaviour was counted during the recording, minimum was 0 (not counted) and without a pre-defined maximum. ^c^: Higher scores indicate better MI-adherence. ^d^: Higher scores indicate more MI non-adherence, and consequently, lower scores indicate better MI-adherence. ^e^: ratio between the number of reflections provided and the number of questions asked. ^f^: Percentage of complex reflections of the total number of reflections (simple and complex). * and **: significantly different from the score in participants where the aim was to change behaviour, with *: *p* < 0.05 and **: *p* < 0.01 (two-sided, not corrected for multiple comparisons).

## Data Availability

Data on motivational interviewing cannot be shared due to privacy regulations. Other data will be made available upon completion of the RCT, and data availability statement will be included in the publication of the RCT. Until the RCT is completed, data cannot be made available.

## Supplementary Materials

The following supporting information can be downloaded at: https://www.mdpi.com/article/10.3390/jcm14113635/s1, Supplementary Material S1: DIASSIST protocol structured education; Supplementary Material S2: DIASSIST protocol motivational interviewing; Supplementary Material S3: Assessment of behavioural components.

## Abbreviations

The following abbreviations are used in this manuscript:

RCT	Randomized Controlled Trial
MI	Motivational Interviewing
FF–TT	Fragile Feet–Trivial Trauma
MITI	Motivational Interviewing Treatment Integrity
COM-B	Capability, Opportunities, Motivation-Behaviour
