# Supplementary material for: Short-Term Efficacy of a Multi-Modal Intervention Program to Improve Custom-Made Footwear Use in People at High Risk of Diabetes-Related Foot Ulceration
Source: J Clin Med. 2025 May 22;14(11):3635. doi: 10.3390/jcm14113635 (PMC12155699; doi:10.3390/jcm14113635)
Supplement: Supplementary file 1 [file jcm-14-03635-s001.zip › Supplementary material S1 - DIASSIST Protocol structured education.pdf]

Protocol education sheet randomization

I would like to explain to you, using the images on this sheet, what we are going to do in this study, and why we are going to do it.

Fragile Feet
Because you have diabetes, your feet are more fragile than feet of most people. This is because diabetes has affected your nerves and blood vessels, among other things. As a result, there are a number of characteristics that you may experience.

For example, you may have:
- Callus formation under your feet
- Dry skin or fissures in your feet
- Discolored skin that feels warm
- Reduced or no feeling in your feet, also known as neuropathy
- Stiffness in your feet and joints, such as in your toes and ankles
- Pain

What do you recognize in these characteristics?

Trivial Trauma
In addition to having more fragile feet, we also see that you have high pressure under your feet when you are walking. When you walk barefoot, the pressure at the bottom of your feet is really high, as you can see in this example. We also see this when walking in regular shoes. These shoes do not specifically target your foot and the places that give you high pressures. The high pressure may lead to trivial trauma.

Is this clear to you so far? Do you understand these images?

Foot ulcer
This combination of your fragile feet and the trivial trauma from excessively high pressures can lead to a foot ulcer. Especially since you don't feel that something is happening under your feet. So, these upper two orange squares represent risk factors for getting such a foot ulcer.

You have had an ulcer before, so you also know what a burden this is and what is required to heal it. The aim of this study is to prevent a new foot ulcer.

How can you achieve this? How can you minimize the risk of a wound? For that, I will go to the bottom two green squares. I will first go back to the fragility of your feet.

Fragile Feet: daily self-care
As I said earlier, your diabetes makes your feet more fragile than most people's, and, unfortunately, we cannot make your feet less fragile. However, you can ensure that they do not become even more fragile by taking good care of them.

Would you mind if we briefly go through this care with you? Some of these actions are things you are already doing, some of them are extra and part of this study.

The advice for your foot care is to perform the following actions daily:

- Check your feet, to see if you find any noticeable spots or changes on your feet

- Measure your foot temperature; this is one of the components of the study and I will discuss this with you later
- Wash your feet well
- Then dry your feet well again; also pay attention to drying between the toes
- Apply cream or emollients to your feet so that the skin remains supple and becomes less dry

In addition, it is advisable to visit the pedicure and podiatrist regularly so that they can give your feet extra care. It is also important to cut your nails properly. Preferably have this done by the pedicure.

Do you have any questions about this?

Preventing Trivial Trauma: shoes and activity
The next thing you can do is to reduce the pressure under your feet. We cannot change the pressure under your bare feet or while wearing socks. As I have just told you, these pressures are very high and the advice is therefore not to walk barefoot or in socks. This is something that a lot of people have already told you, but is very important to keep in mind.

But what can you do? You have custom-made footwear. This is specially made for your feet to keep the pressure points under your feet as low as possible. By always walking in your shoes, you relieve your feet of the high pressure.

We measured your shoes during the previous visit and we saw: [*select answer depending on findings*]

a) That your shoes are well-adjusted to your feet and do not need any further adjustments at this time. It is good to repeat the measurements regularly because your feet can also change. That is also the reason why we have measured this now.
b) That your shoes still gave (slightly) too high pressure. Based on these measurements, the shoe technician is now working on a number of adjustments and as soon as these are ready we can measure the shoes again.

When the participant does not yet have indoor footwear, continue with:
Furthermore, we know that it may be difficult to always wear your shoes, especially indoors. That is why we want to offer you a custom-made indoor shoe, in addition to your current custom-made shoes. We know from previous research that this method works very well to improve indoor shoe use and we have also heard from these participants that it is very nice to have a separate shoe for in the house. Are you interested in this?

When the participant already has indoor footwear, continue with:
You already have a pair of custom-made indoor footwear.

In addition, you also see an image here indicating that you should reduce your activity when you find a hotspot or abnormal spot. This has to do with the temperature measurements I mentioned earlier. I will explain them to you later and then I will come back to this. The most important thing is to remember that when something looks different or when you measure something different than normal, you should reduce your activity to give your feet some time to rest.

No foot ulcer
By performing these actions, we try to keep the risk of a new wound as low as possible. This is therefore a combination of good care and relieving the burden on your feet.

Do you have any questions about this?

At the end of the visit
I have explained you a lot of things today, and we have been through the extra actions you will do because of the study. Before we end here today, I want to come back to the sheet again.
Can you summarize what I have told you, but in your own words?
