# Supplementary material for: Short-Term Efficacy of a Multi-Modal Intervention Program to Improve Custom-Made Footwear Use in People at High Risk of Diabetes-Related Foot Ulceration
Source: J Clin Med. 2025 May 22;14(11):3635. doi: 10.3390/jcm14113635 (PMC12155699; doi:10.3390/jcm14113635)
Supplement: Supplementary file 1 [file jcm-14-03635-s001.zip › Supplementary material S2 - DIASSIST protocol motivational interviewing.pdf]

Protocol Motivational Interviewing DIASSIST

Reading guide: normal text, is researcher's text. Italics provide guidance or background. Dots/text between square brackets (e.g.: [...]) is where the participant says something.

First MI-session

Phase 1: Engage

Thank the participant for speaking with you on the telephone. Explain the reason for the chat (see phase 2).

Good day, nice that speak with each other.

Have a short chat about general aspects, to engage.

Phase 2: Focus

As discussed with you during your previous visit for the DIASSIST study, we have this conversation to talk about preventing wounds and wearing orthopedic shoes. The researcher explained last time how preventing wounds and wearing shoes are related. What do you remember about that?

Step 1a: If the participant remembers anything, reinforce this positively. Then zoom in on wearing orthopedic shoes.

Indeed, you remembered that well. [*Reinforce what was said*]. Repeat what DIASSIST is about (shoe and temperature) once more.

Step 1b: If the participant does not know anything or very little anymore, ask permission to explain it again – and explain.

I understand that. There was a lot of information provided last time. Do you mind if I explain it again? [Yes]

Explain about fragile feet – trivial trauma, and the importance of wearing custom-made footwear at all times.

When discussing footwear, use sentences like: "we know from previous research that wearing custom-made footwear at all times reduce the risk of getting a new foot ulcer."

Ask if all is clear, ask participant to summarize.

As discussed during your previous visit for the DIASSIST study, one aim of this conversation is to discuss the wearing of your custom-made footwear. We have measured this as well. Are you ok with discussing this in more detail?

[Yes].

If yes, move to phase 3. If no, talk about it.

Phase 3: Elicit

As I just said, we have measured your wearing of your custom-made footwear. The next step is to show and discuss this. I am curious about your thoughts about this. Are you ok with this?

When wearing time is <8 hours/day, the aim is to motivate to change this behaviour.

When wearing time is >8 hours/day, the aim is to sustain this healthy behaviour, and potentially to motivate to increase this even further.

Your wearing time is [xxx]. When you hear this, what do you recognize / think? [...]
Provide reflection.

When wearing time is high, give a compliment. Use sentence structures like:

Indeed, your wearing time is (very) high, which is great. We do not see many people reaching such high wearing times. Well done. How did you achieve this?

When wearing time is low, try to elicit 'change talk'.

First, ask permission to provide information, then ask eliciting questions.

Can I tell you something more about people like you, and what they experience when they wear their custom-made footwear more frequently? [Yes]

As I said before, when you wear your custom-made footwear at all times, you reduce the risk of getting a new foot ulcer. Preventing a foot ulcer also means not having to go to hospital, and being able to do the things you like. What do you recognize from that?

Eliciting questions

For each question the following applies:

- *reflect and specify ambivalence – then reinforce positively – then new open question to elicit change talk*
- *you can strategically provide a reflection that is slightly incorrect, to elicit a reaction and thus change talk*
- *if your tone goes down at the end it is a reflection*
- *you listen, you give feedback with objective results, you do not enter into discussion*
- *compliment good behavior and good efforts*

Wherever the protocol says "wearing your orthopedic shoes" it can also say "measuring your foot temperature", if that is the subject. If the goal is to sustain healthy behaviour, then everywhere where it says "change" change to "continue like this".

Preparatory questions

<u>Desire to change:</u>

- What is not going well now about wearing your orthopedic shoes, that you would like to change?
- What problems would you like to see disappear, if you choose to wear your orthopedic shoes more?
- What complaints do you have now, that you do not want to suffer from anymore?
- What would you like to see differently in terms of your feet or wearing of your shoes, a year from now, when the study has ended?

<u>Disadvantages of current behaviour:</u>

- What problems do you experience when you wear your orthopaedic shoes less often?
- What disadvantages have you already experienced from wearing your orthopaedic shoes less often?
- What are the 3 most important disadvantages of wearing your orthopaedic shoes less often?
- What are you not doing at the moment, for fear of new foot ulcers?
- How have your experiences (fill in based on the previous points – related to ulceration) prevented you from doing what you would like to do?
- What obstacles have you experienced from wearing your shoes less often?

<u>Advantages of desired behaviour:</u>

- Suppose you choose to wear your shoes more often, what will this bring to you?

- Suppose you choose to wear your shoes more often, which problems will decrease?
- What are the 3 best reasons to wear your shoes more often?
- What concerns do you take away from those around you if you start wearing your shoes more?
- Suppose you choose to wear your shoes more, what will you gain from that?

<u>Capacity for change:</u>

- On some days you wore your shoes a lot, more than 80% of your steps / more than 8 hours on a day. How did you do that?
- What is important to you, which would enable you to wear your shoes all day?
- Who can support you in wearing your orthopedic shoes more?
- What qualities do you have that enable you to wear your shoes more?
- What qualities do you have that can help you wear your shoes?
- What have you already done to wear your shoes more?
- How would you approach wearing your shoes more?
- Who can help you wear your shoes more?
- You indicate that on some days you just didn't feel like wearing them at all times / during all steps. On a scale of 1 to 10, how confident are you that you can wear your shoes more?
    - Positively reinforce the number
    - What makes you give yourself an X, and not an X-1?
    - What needs to happen to go from an X to an X+1 or even an X+2?

<u>Need to change:</u>

- How important is it to you to wear your shoes all day?
- Let's just leave the how for now. What do you think needs to change in wearing of your shoes?
- On a scale of 1 to 10, how important is it to you to wear your shoes more?
    - Positively reinforce the number
    - What makes you give yourself an X, and not an X-1?
    - What needs to happen to your complaints to go from an X to an X+1 or even an X+2?

<u>Ask about extremes:</u>

- What is the worst that can happen if you don't wear your shoes more?
- What is the best thing that you will be able to do again if you wear your shoes more?
- If all the changes you want were to succeed, what would your foot and your life look like?

Looking back:

- You got a wound because you didn't wear your shoes very often. What could you have done differently then?
- You have previously shown that you can wear your shoes a lot. How did you do that then?
- Can you remember a time when you wore your shoes more often? [Yes]. What has changed?
- How have the wounds you had on your feet changed you as a person?
- How have the wounds you had on your feet changed the way you wear your shoes?

Looking ahead

- Suppose you decide to wear your shoes more often, what do you hope to experience differently in the future?
- Suppose you don't change anything, what will your life look like in 5 years?

Mobilizing questions

Key questions

- If you hear all this from yourself, what is the next step?
- What do you think you are going to do now with regard to wearing your shoes?
- How are you going to do it the same way again next week?

Action

- How ready are you to wear your shoes more?
- How willing are you to try to wear your shoes more?
- What steps are you willing to take this week to wear your shoes more?

Commitment

- What are you going to do?
- Tell me what you are going to do about wearing your shoes?
- What are you planning to do?
- Tell me what your plans are for wearing your shoes?

Phase 4: Planning

Commitment has been shown – time to plan the behavior change (or the sustaining of the healthy behaviour).

It's really great that you want to do that. Can I explain why it is useful to convert that into a plan? [Yes]

What we know is that people are more likely to maintain a behavior change if they have written it down. Maintaining behavior is terribly difficult. Just think of the dentist, and the promise to clean. That is why I want to help you wear your shoes more. That is why I want to make a plan with you. What do you think when you hear that? [...]

The plan includes:

- Your goal. What is your goal? [...]
- The date: When are you going to start with that? [...]
- Until when: How long do you want to do that? [...]
- Measures that help you:
  - Avoidance
    Activities, places, or people that I will avoid in the coming period are:
  - Behavioral alternatives
    Alternative behavior at times when I do not want to wear my shoes:
  - Rewards:
    If I achieve my goal, my rewards are:

<u>Follow-up questions:</u>

- Just to be sure: what exactly have we agreed on?
- What are you going to do? Can you write it down?
- When will you start with this?
- How are you going to approach doing it differently?
- What will you do at times when there is a high probability that you will not be wearing the shoes?
