# Supplementary material for: Short-Term Efficacy of a Multi-Modal Intervention Program to Improve Custom-Made Footwear Use in People at High Risk of Diabetes-Related Foot Ulceration
Source: J Clin Med. 2025 May 22;14(11):3635. doi: 10.3390/jcm14113635 (PMC12155699; doi:10.3390/jcm14113635)
Supplement: Supplementary file 1 [file jcm-14-03635-s001.zip › Supplementary material S3 - assessment of behavioural components.pdf]

Supplementary S3: Assessment of the behavioural components included in each modality of the intervention

	**Structured education**	**Motivational interviewing**	**Custom-made indoor footwear**
**Behaviour source**			
Capability – Psychological			
Capability – Physical			
Opportunity – Social			
Opportunity – Physical			
Motivation – Automatic			
Motivation - Reflective			
**Intervention functions**			
Education			
Persuasion [a]			
Incentivisation			
Coercion			
Training			
Enablement			
Modelling			
Environmental restructuring			
Restrictions			
**Behaviour change techniques** [b]			
1.1 Goal setting (behaviour)			
1.2 Problem solving			
1.3 Goal setting (outcome)			
1.4 Action planning			
1.5 Review behaviour goal(s)			
1.6 Discrepancy [...]			
1.7 Review outcome goal(s)			
1.8 Behavioural contract			
1.9 Commitment			
2.2 Feedback on behaviour			
2.7 Feedback on outcome(s) of behaviour			
5.1 Information about health consequences			
5.2 Salience of consequences			
8.2 Habit formation			
8.3 Habit reversal			
9.2 Pros and cons			

9.3 Comparative imaging of future outcomes			
15.2 Mental rehearsal of successful performance			
15.3 Focus on past success			
**Mechanism of action**			
Knowledge			
Beliefs about capabilities			
Beliefs about consequences			
Intention			
Goals			
Environmental context and resources			
Behavioural regulation			
Attitudes toward the behaviour			
Motivation			
Feedback processes			
Behavioural cueing			
Perceived susceptibility/vulnerability			

Note: the shaded cells indicate that this behavioural component is included in the modality. The darkness of the shade indicates the frequency with which this component is present, based on qualitative assessment of the authors. [a]: According to the protocol and the spirit of MI, persuasion should be done with permission. However, this also occurred without permission (see results). Therefore, the general intervention function ‘persuasion’ is indicated here. [b]: only behaviour change techniques and mechanisms of actions that were identified are shown in the table, not all 74 techniques and 26 mechanisms.
